# Mechanisms of Trichoderma–Mediated Plant Growth Promotion: From Metabolites to Plant Responses

**DOI:** 10.3390/plants15142180

**Published:** 2026-07-16

**Authors:** Juan Francisco Jiménez Bremont, Mayra Santiago Velasco, María de los Ángeles Bohórquez Quintero, Enrique González Pérez, Margarita Rodríguez-Kessler

**Affiliations:** 1 División de Biología Molecular, Instituto Potosino de Investigación Científica y Tecnológica (IPICYT), San Luis Potosí 78216, Mexico; mayra.santiago@ipicyt.edu.mx (M.S.V.); maria.bohorquez@ipicyt.edu.mx (M.d.l.Á.B.Q.); enrique.glez6891@gmail.com (E.G.P.); 2 Facultad de Ciencias, Universidad Autónoma de San Luis Potosí (UASLP), Av. Chapultepec 1570, Priv. del Pedregal, San Luis Potosí 78295, Mexico; margarita.rodriguezydominguez@uaslp.mx

**Keywords:** biostimulants, nutrient acquisition, plant growth promotion, plant-microbe interactions, phytohormone modulation, sustainable agriculture, *Trichoderma*

## Abstract

The transition toward sustainable agriculture has driven the use of plant growth-promoting microorganisms as viable alternatives to conventional agrochemicals. Among these, species of the genus *Trichoderma* have emerged as pivotal players due to their ability to establish beneficial and complex interactions with plants that extend far beyond their traditional role as biocontrol agents. This review integrates recent advances in understanding the mechanisms by which *Trichoderma* promotes plant growth and development. Specifically, the evolutionary transition of *Trichoderma* from a mycoparasitic lifestyle to beneficial plant associations is examined, together with the molecular processes involved in plant recognition, root colonization, and establishment within the rhizosphere and plant tissues. Furthermore, the mechanisms through which *Trichoderma* enhances plant performance are addressed, including metabolite production (both volatile and non-volatile), phytohormone modulation, nutrient acquisition and mobilization, and enhanced photosynthetic capacity. Finally, current agricultural applications, the technical challenges of formulation and field implementation, and future perspectives on *Trichoderma*-based microbial consortia and precision agriculture tools are analyzed. Overall, this review provides a comprehensive framework highlighting the potential of *Trichoderma* as a premier biostimulant for modern sustainable farming.

## 1. Introduction

### 1.1. Plant Growth-Promoting Microorganisms and Their Importance in Agriculture

Modern agriculture faces growing pressure to enhance crop yields while simultaneously reducing the environmental footprint of intensive chemical fertilizer and pesticide use. In this context, plant growth-promoting microorganisms (PGPMs) have emerged as an effective biotechnological strategy for improving crop productivity and to develop more resilient and environmentally sustainable agricultural systems [1,2,3]. Among these microorganisms, bacteria of the genera *Pseudomonas*, *Bacillus*, and *Streptomyces*, as well as filamentous fungi such as *Trichoderma* and mycorrhizal fungi, are known to colonize the rhizosphere and plant tissues, establishing mutualistic interactions that promote plant growth and development. These PGPMs stimulate plant growth through direct and indirect mechanisms, including phytohormone production (auxins, cytokinins, and gibberellins), biological nitrogen fixation, mineral nutrient solubilization (particularly potassium and phosphorus), siderophore secretion for iron acquisition, and the emission of volatile organic compounds (VOCs) that modulate plant growth and defense pathways [1,4,5]. Additionally, PGPM-based bioinoculants enhance plant tolerance to biotic and abiotic stresses by improving nutrient acquisition, producing bioactive compounds, and activating induced systemic resistance (ISR), thereby increasing plant resilience [6]. Consequently, they constitute a key strategy for reducing agrochemical inputs, restoring soil health, and promoting sustainable agriculture.

Among PGPMs, *Trichoderma* species are particularly noteworthy for their multi-functional traits. Their capacity for efficient rhizosphere and root colonization, coupled with biostimulant and biofertilizing activities, induction of systemic resistance, enhanced tolerance to abiotic stress, and broad-spectrum antagonism against phytopathogens, makes them among the most versatile microbial agents for sustainable agriculture [7,8].

### 1.2. Trichoderma as a Beneficial Fungus in Sustainable Agriculture

The genus *Trichoderma* comprises saprophytic fungi characterized by rapid mycelial growth, high colonization capacity, competitive ability in soil ecosystems, and remarkable adaptability to diverse environmental conditions [9,10]. Notably, the beneficial effects of *Trichoderma* fungi are highly strain-specific. Accumulating evidence demonstrates that different *Trichoderma* species, and even distinct isolates within the same species, exhibit considerable variation in their capacity to colonize plant roots, synthesize bioactive metabolites, induce plant defense responses, and promote plant growth [8,11,12].

*Trichoderma* species serve as effective biocontrol agents against plant phytopathogens. By competing for resources and colonizing intercellular root spaces, they successfully limit pathogen invasion [10,13]. Furthermore, *T. atroviride* expresses ABC transporters such as *Taabc2*, which has been associated with detoxification processes, tolerance to inhibitory compounds, and biocontrol activity. These transporters may contribute to fungal survival and competitiveness in the chemically complex rhizosphere environment [14].

Beyond their established role as biocontrol agents, *Trichoderma* species form intimate, beneficial associations with plant roots. Although traditionally regarded as rhizosphere-restricted symbionts [10,15], recent evidence confirms their capacity to establish true endophytic associations [7,16]. This lifestyle spectrum is highly strain-dependent, ranging from aggressive mycoparasitism to facultative endophytism [7,17]. Mechanistically, comparative genomic studies have revealed that endophytic strains possess a greater diversity of biosynthetic and degradative gene clusters than their non-endophytic counterparts, which may facilitate their adaptation to the internal plant environment [7,18].

The *Trichoderma*–plant interaction confers multiple benefits, most notably promoting plant growth and development [19,20,21]. These advantages are driven by complex mechanisms, including mycoparasitism, competition for nutrients and space, antibiosis mediated by secondary metabolites, and the induction of systemic plant resistance [10,20,21]. Due to their high nutritional adaptability, *Trichoderma* species can colonize a wide range of ecological niches [9]. Moreover, several *Trichoderma* species and strains can colonize a wide variety of plant hosts [10,13], a key factor in their selection as biocontrol agents and biostimulants. *Trichoderma* species also promote plant vigor by facilitating nutrient uptake, secreting and/or inducing the biosynthesis of phytohormones, and improving plant tolerance to abiotic stresses [20,22,23]. Altogether, these multifaceted functions position *Trichoderma* as a key microbial genus for the development of sustainable agricultural strategies that enhance plant growth while reducing dependence on chemical inputs.

Given these diverse beneficial functions and the growing interest in the research and application of *Trichoderma*, this review aims to examine its role as a plant growth-promoting fungus and to highlight the mechanisms underlying its beneficial effects on plant growth and development. It also addresses the evolutionary transition of *Trichoderma* from an ancestral mycoparasite to a plant-associated symbiont, including the processes involved in root colonization and the establishment of symbiosis. In addition, it explores metabolite production by *Trichoderma*, phytohormone modulation, and molecular signaling involved in plant–fungus communication, as well as *Trichoderma*’s contribution to nutrient acquisition and plant development. Finally, its use in agriculture as a biostimulant is discussed, along with current challenges and future perspectives for sustainable agricultural management.

### 1.3. Evolution of Trichoderma Species: From Ancestral Mycoparasite to Plant Symbiont

Based on genomic and phylogenomic analyses, mycoparasitism has been proposed as the ancestral lifestyle of the genus *Trichoderma* [24]. However, some species, such as *T. reesei*, exhibit gene loss associated with this capacity, reflecting their adaptation to a predominantly saprotrophic lifestyle specialized in degrading plant biomass [24,25,26]. In this context, these fungi likely interacted with plants as an evolutionary strategy to access ecological niches rich in potential prey, particularly phytopathogenic fungi inhabiting the rhizosphere. Under this model, *Trichoderma*, by feeding on other fungi, may have initially acted as an indirect biocontrol agent protecting plants. This scenario likely facilitated the transition toward closer and more coordinated interactions with plants, allowing the genus to evolve into the symbiotic and endophytic associations observed today. Consistent with this evolutionary perspective, phylogenetic studies have shown that the genus *Trichoderma* has undergone multiple shifts in habitat preference and nutritional modes, including transitions among saprotrophic decomposers, mycoparasites, and plant-associated symbionts [24,27].

In addition to their mycoparasitic activity, *Trichoderma* species exhibit remarkable nutritional versatility, as they can act as saprotrophs capable of degrading plant biomass and, in some cases, interact with insects, reflecting their extensive enzymatic and metabolic repertoire [17,24,28]. This ecological plasticity helps explain the genus’s wide distribution across diverse ecosystems and its ability to interact with different hosts.

Consistent with this ecological diversity, recent genomic analyses have revealed notable genetic plasticity within the genus *Trichoderma*. In particular, Mondal et al. [29] reported that the genus possesses an open pangenome, characterized by a core set of genes associated with essential functions and a large repertoire of accessory genes involved in ecological adaptation. This genomic architecture helps explain functional diversification among species, with some specializing in the production of lignocellulolytic enzymes of industrial interest, while others exhibit expansions in genes related to secondary metabolism and biocontrol-associated mechanisms [29].

## 2. Root Colonization and Symbiotic Establishment of Trichoderma

### 2.1. Recognition and Recruitment in the Rhizosphere

**Figure 1:**
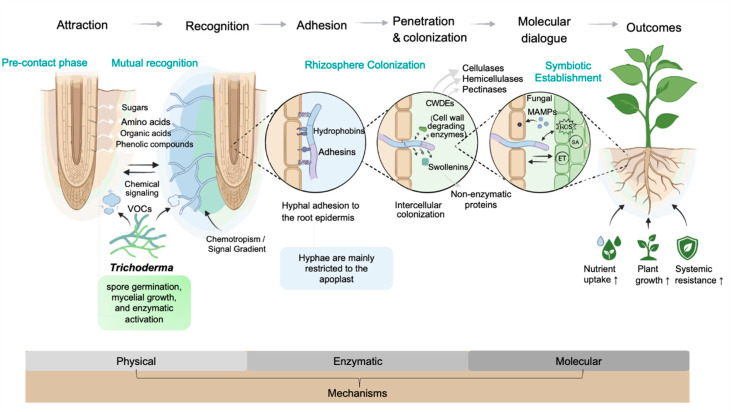
Mechanisms of rhizosphere colonization and establishment of beneficial interaction between *Trichoderma* and plants. During the pre-contact phase, volatile organic compounds (VOCs) and root exudates promote fungal attraction, spore germination, and mycelial growth. During the recognition and colonization phase, *Trichoderma* adheres to the root epidermis through the action of hydrophobins, adhesins, swollenins, and cell wall-degrading enzymes (CWDEs). A molecular dialogue mediated by VOCs, other fungal metabolites, phytohormones such as salicylic acid (SA) and ethylene (ET), and reactive oxygen species (ROS) modulates plant responses associated with growth, development, and defense. Colonization is restricted primarily to the apoplast, where the fungus promotes nutrient absorption, plant growth, and induced systemic resistance (ISR). The upward arrows (↑) indicate an increase in the corresponding biological process or physiological response. Illustration generated with FigureLabs and subsequently modified and validated by the authors for scientific accuracy.

The *Trichoderma*–plant interaction is a dynamic process that begins with fungal attraction, recognition, and growth toward the root, followed by rhizosphere colonization and the establishment of an early molecular dialogue [30,31]. Figure 1 presents a schematic representation of the mechanisms involved in establishing the *Trichoderma*–plant interaction during rhizosphere colonization and symbiotic establishment. The pre-contact phase is mediated by diffusible and volatile chemical signals that enable mutual recognition between the fungus and the plant [31,32,33]. During precolonization, a bidirectional exchange of metabolites occurs, in which fungus- and plant-derived compounds coordinate early physiological and molecular responses [30,31]. Volatile organic compounds (VOCs) produced by *Trichoderma* act as long-distance signaling molecules, including terpenes and lactones such as 6-pentyl-α-pyrone, while other diffusible metabolites, such as auxins, organic acids, and polyketides, contribute to recognition and the modulation of initial physiological responses associated with plant biostimulation [12,22,32,34].

Concurrently, roots release a diverse array of exudates that establish the chemical niche of the rhizosphere, including sugars, amino acids, organic acids, and phenolic compounds (Figure 1). These compounds function not only as nutrient sources but also as key determinants of rhizosphere microbial assembly and signaling, modulating the microbial community composition and the ecological dynamics of the root environment [31,35]. In *Trichoderma*, root exudates stimulate essential physiological responses, such as spore germination, mycelial growth, and the activation of key enzymatic pathways [36,37], all of which are necessary for successful colonization. Certain metabolites, such as sucrose, facilitate the initial recruitment and attraction of *Trichoderma* to the root surface. *T. atroviride* modifies the composition of root exudates by increasing sucrose availability, thereby promoting fungal growth and influencing rhizosphere interactions [38]. Similarly, *T. virens* utilizes host-derived sucrose as a carbon source during root colonization, thereby modulating plant metabolism and promoting the establishment of the plant–fungus interaction [39]. Furthermore, root exudates from stressed plants contain defense-related compounds, including plant-derived proteins like peroxidases, that may contribute to the attraction, chemotaxis, and stimulation of *Trichoderma* growth [36,37].

Signaling molecules such as the oxylipin 13-(S)-HODE act as potent chemoattractants, triggering directed hyphal growth toward the root through finely tuned positive chemotropic responses to plant-derived signals [40]. This perception guides mycelial growth toward metabolically active zones, including the elongation region and root hairs [30,41]. Following initial contact, the fungal secretome plays a key role in root adhesion and the establishment of successful colonization [30,42].

### 2.2. Establishment of Trichoderma in the Root

Following entry into root tissues, *Trichoderma* colonizes the epidermis and outer cortical layers. Hyphae are primarily restricted to the apoplast, growing within these tissues without penetrating the inner cortex or the vascular system [43,44,45]. This restricted colonization is largely associated with the activation of the plant immune system, including cell wall reinforcement through callose deposition, and the production of reactive oxygen species (ROS), ethylene (ET), and antimicrobial secondary metabolites [9,45,46,47]. In this context, salicylic acid (SA) plays a central role in limiting fungal invasion and restricting colonization to the outer root layers [48].

Concurrently, JA/ET and SA signaling pathways are finely coordinated during the interaction to maintain the beneficial association while priming plant defenses [49,50]. This hormonal balance is associated with the transcriptional reprogramming of WRKY transcription factors, which integrate hormone signaling and defense responses during root colonization [51]. In particular, the induction of *WRKY33* by *Trichoderma* has been proposed to contribute to the modulation of SA signaling, thereby facilitating the establishment of a prolonged symbiotic association [49].

The activation of ISR by *Trichoderma* begins with the perception of fungal microbe-associated molecular patterns (MAMPs), including hydrophobins, cerato-platanins, swollenins, xylanases, cellulases, and chitin-derived oligosaccharides, by plant pattern recognition receptors (PRRs) [30,42,47,52]. Recognition of MAMPs by PRRs triggers basal immune responses characteristic of pattern-triggered immunity (PTI), including transient Ca^2+^ influx, ROS production, MAPK activation, and defense-related transcriptional reprogramming [53,54,55]. Rather than inducing constitutive defense responses, these early signaling events establish a primed state that enables a faster and stronger defense response upon pathogen attack, while maintaining compatibility with beneficial root colonization by *Trichoderma* [21,30,53,56]. Thus, *Trichoderma* actively modulates host immunity by reprogramming the plant transcriptome and secretome, suppressing specific plant responses such as reduced secretion of defense-related proteins (glycosyl hydrolases and peroxidases), and active ROS scavenging, thereby favoring the establishment and maintenance of a compatible symbiotic interaction [30,56,57,58].

This regulation of plant immunity allows controlled root colonization without compromising the establishment of ISR and a primed defense state. For instance, *T. asperelloides* suppresses nitric oxide (NO) production elicited by *Fusarium oxysporum* and modulates the expression of NO-responsive genes, demonstrating its capacity to modulate early defense signaling pathways in plants [46]. Therefore, these mechanisms contribute to the establishment of a compatible interaction while maintaining the plant in a primed state for enhanced defense against subsequent pathogen attacks, highlighting the beneficial role of *Trichoderma* in plant health and disease resistance [55,59,60].

### 2.3. Mechanisms of Root Colonization by Trichoderma: Adhesion, Penetration, and Molecular Dialogue

#### 2.3.1. Adhesion Mechanisms: Hydrophobins and Adhesins

Upon contact with the root surface, *Trichoderma* activates a series of mechanical and enzymatic processes that facilitate anchoring and subsequent tissue penetration. Central to this initial phase is the deployment of adhesion proteins, specifically hydrophobins and adhesins, which mediate stable attachment to the rhizoplane (Figure 1) [30,61,62]. This attachment is a prerequisite for successful colonization, as it stabilizes the fungus–host interface during the subsequent stages of establishment [10,62].

Adhesins are key proteins involved in the recognition and anchoring of *Trichoderma* to the rhizoplane [61]. These cell-surface proteins facilitate the attachment of both conidia and hyphae through interactions with host-derived glycans, glycoproteins, and mucilage polysaccharides, thereby establishing the stable adhesion required for successful colonization [30,61]. While fungal adhesins are commonly classified into the PA14, Flo11, and Als families, their characterization in *Trichoderma* remains largely computationally predicted. Genomic analyses have identified putative adhesins based on homology with well-characterized orthologs, including Mad1 and Mad2 from *Metarhizium* and Fas1 and Wsc1 from *Verticillium* [61]. Nevertheless, these candidate proteins still lack functional validation, highlighting a critical gap in our understanding of the molecular mechanisms governing *Trichoderma* root adhesion.

Hydrophobins, small cysteine-rich proteins unique to filamentous fungi, are important because they self-assemble into amphipathic layers at hydrophilic–hydrophobic interfaces. This molecular reorganization effectively modifies the surface of fungal hyphae and conidia, facilitating fungal attachment and colonization of the root surface [62,63]. Studies using mutants in hydrophobin-encoding genes consistently show reduced root adhesion and colonization, highlighting the contribution of hydrophobins during the early stages of fungal establishment [62,64,65]. For instance, in *T. virens*, the hydrophobin TVHYDII1 enhances both root colonization and antagonistic activity, whereas deletion of this gene severely compromises these capacities [64]. HFB9A has been identified as a key factor in colonization, as *Δhfb9a* mutants exhibit reduced fungal establishment, decreased cell wall-degrading enzyme activity, and inability to induce systemic resistance against *Colletotrichum graminicola* [65]. In addition, *TasHyd1* mutants of *T. asperellum* also exhibit reduced adhesion and root colonization [62]. Beyond their role in adhesion, the hydrophobin HYTLO1 from *T. longibrachiatum* functions as a MAMP that triggers ISR while simultaneously promoting root growth [66].

Although hydrophobins and adhesins contribute to root adhesion, this process has been proposed to be multifactorial, involving several surface-associated proteins [47,61]. However, future functional studies of adhesins are required to determine whether these proteins exhibit complementary roles or potential redundancy with hydrophobins during the adhesion process.

#### 2.3.2. Cell Wall Remodeling and Secreted Proteins During Root Colonization

Following adhesion, *Trichoderma* initiates a controlled penetration into the outer root tissues, generally restricted to the epidermis and outer cortex [44]. This penetration occurs through the coordinated secretion of CWDEs, such as cellulases, hemicellulases, and pectinases, which locally modify the polysaccharide matrix and facilitate hyphal progression without causing extensive tissue damage [30,67]. Beyond their role in cell wall remodeling, CWDEs, which constitute a significant fraction of the *Trichoderma* secretome, also contribute to signaling during the interactions with plants [30]. Some of these secreted proteins, such as xylanases and cellulases, have been described as MAMPs, as they can be recognized by plant receptors and trigger early immune responses [30,68,69]. On the other hand, the activity of these enzymes can generate oligosaccharides derived from the plant cell wall that function as damage-associated molecular patterns (DAMPs). For example, Morán-Díez et al. [70] reported that the endopolygalacturonase ThPG1 from *T. harzianum* is involved in the release of oligogalacturonides, which act as signaling molecules during the plant–fungus interaction. Altogether, this restricted colonization strategy favors the establishment of a symbiotic association with the host plant (Figure 1).

In addition, non-enzymatic proteins such as swollenins, which are structurally similar to plant expansins, contribute by loosening cellulose and facilitating mycelial advancement [67,71]. Studies in *T. guizhouense* have shown that the TgSWO protein modifies cell wall architecture and promotes plant growth [72], suggesting that swollenins not only participate in the initial penetration process but also modulate plant–fungus interactions.

Cysteine-rich cell wall proteins also participate in the plant–fungus interaction. A well-characterized example is the protein encoded by the *qid74* gene in *T. harzianum*, which modifies root architecture by stimulating lateral root formation and root hair proliferation. This morphological change enhances nutrient uptake and significantly increases plant biomass [73]. Another important group is the cerato-platanin family, which comprises small secreted cysteine-rich proteins that act as elicitors of ISR. Among these, Sm1 from *T. virens* and its ortholog, Epl1 from *T. atroviride*, are among the best-characterized proteins [74]. *Arabidopsis* lines expressing *Epl1* from *T. atroviride* exhibit enhanced growth and increased resistance against the necrotrophic fungus *Botrytis cinerea* and the hemibiotrophic bacteria *Pseudomonas syringae* pv. *tomato* DC3000, a phenotype associated with elevated ROS accumulation and activation of systemic defense pathways [75]. However, further research is needed to elucidate the specific molecular pathways through which this elicitor promotes plant growth.

#### 2.3.3. Coordinated Molecular Dialogue During Root Colonization

Root colonization by *Trichoderma* involves a coordinated molecular dialogue involving physical interactions, enzymatic activities, and molecular signaling (Figure 1). Proteomic studies of the interaction between *T. guizhouense* and cucumber roots have revealed that early colonization induces a dynamic modulation of the fungal proteome in response to root exudates, thus promoting plant growth, a process associated with auxin production and nitrogen metabolism. In addition, the secretion of carbohydrate-active enzymes, including glycoside hydrolases and β-glucosidases, facilitates root penetration and colonization [67].

Taken together, these coordinated events, from adhesion mediated by hydrophobins and adhesins to restricted penetration facilitated by CWDEs and swollenins, allow the fungus to colonize the root in a controlled manner [30,45,61]. Concurrently, the secretion of effectors and elicitors, such as cerato-platanins, together with the modulation of plant metabolism and phytohormone signaling, regulates host responses to activate induced defenses [42,52,67]. Overall, these responses contribute to a beneficial plant–fungus interaction, promoting plant growth and enhancing tolerance to biotic and abiotic stresses.

## 3. Metabolites of Trichoderma and Their Role in Plant Growth Promotion

### 3.1. Trichoderma Volatile Organic Compounds

Numerous studies have demonstrated that volatile organic compounds (VOCs) emitted by *Trichoderma* species induce physiological and molecular responses in different organisms, including plants [32,76]. VOCs are low-molecular-weight organic molecules with high vapor pressure and low boiling points, allowing them to disperse efficiently through soil, water, and air, thereby acting as long-distance signaling molecules in plant-microbe communication [76]. *Trichoderma* VOCs have been shown to promote growth, remodel root architecture, and suppress pathogens without direct physical contact [12,32,77,78].

Over the past decade, studies using gas chromatography–mass spectrometry (GC-MS) have shown that *Trichoderma* species produce complex and diverse VOC mixtures, including alcohols, aldehydes, ketones, esters, aromatic compounds, terpenes, furans, lactones, alkanes, alkenes, sulfur- and nitrogen-containing compounds, among others [11,12,32,79]. The composition and abundance of these profiles depend on the *Trichoderma* species, culture conditions, nutrient availability, and developmental stage [32].

The signaling role of *Trichoderma* VOCs has been demonstrated in several plant species, including Arabidopsis, lettuce, tomato, and maize, where exposure to these fungal volatiles promotes plant growth and modifies root system architecture [11,78,80]. In *Arabidopsis*, exposure to *Trichoderma* VOCs promotes local auxin accumulation in root tissues, as evidenced by increased *DR5* reporter activity [12,32,77,78]. Furthermore, VOCs from different *Trichoderma* species not only enhance shoot and root biomass but also increase chlorophyll content, suggesting a positive effect on photosynthetic performance and plant development [11]. Transcriptomic analyses performed under both non-stress [81] and salt stress conditions [82] revealed that exposure to *Trichoderma* VOCs induces extensive transcriptional reprogramming associated with growth regulation, hormone signaling, cell wall remodeling, and stress adaptation.

For instance, the lactone 6-pentyl-α-pyrone (6-PP), a VOC produced by several *Trichoderma* species [32,83], is characterized by its distinctive coconut-like odor. Several studies have shown that 6-PP promotes plant growth [34,84], an effect associated with hormonal signaling pathways, particularly auxins. Early reports suggested that 6-PP exhibits auxin-like activity [85], and later studies demonstrated that its effects involve components of auxin transport and signaling pathways [34]. In addition, González-Pérez et al. [26] reported that *T. atroviride* produces 6-PP at significant levels, representing 29.33% and 33.86% of total VOCs in MS and PDA media, respectively. During the *Arabidopsis*-*T. atroviride* interaction, the relative abundance of 6-PP was substantially higher in PDA (30.06%) than in MS (1.41%), suggesting that its production is influenced by culture conditions and plant–fungus interactions.

Sesquiterpenes (SQTs) are among the most abundant compounds in *Trichoderma* VOC profiles [11,12,86,87], but their effects on plant physiology remain poorly studied. In this regard, González-Pérez et al. [26] reported that the most frequent and abundant VOCs produced by *T. virens*, both in axenic culture and during interaction with *A. thaliana*, were terpenes, particularly SQTs. Likewise, the SQT cedrene, identified in *T. guizhouense*, promotes root development in *Arabidopsis* through auxin transport and signaling pathways [88].

In addition to SQTs, several individual VOCs within the *Trichoderma* volatilome have been associated with plant growth promotion [78,81]. Specifically, individual compounds such as 3-methyl-1-butanol, 1-decene, and 2-heptylfuran increase fresh weight and chlorophyll content, whereas 1-decene additionally upregulates the expression of genes associated with cell wall modification and auxin signaling [81].

Although individual VOCs exhibit distinct biological activities, their effectiveness likely depends on their composition and relative abundance within complex volatile blends. Consequently, these natural VOC mixtures may function as multifunctional signals that coordinate plant physiological responses and promote growth.

### 3.2. Non-Volatile Metabolites Secreted by Trichoderma and Their Impact on Plant Growth

*Trichoderma* secretes various non-volatile metabolites that directly impact plant growth, including organic acids, siderophores, and other bioactive secondary metabolites that enhance nutrient solubilization and plant development [89]. In particular, organic acids such as gluconic, citric, oxalic, and fumaric acids contribute to phosphate solubilization and rhizosphere acidification, while siderophores improve iron bioavailability under limiting conditions [89,90,91,92]. Additionally, the metabolite harzianolide, isolated from *T. harzianum*, promotes tomato seedling growth and triggers ISR against fungal pathogens [93].

## 4. Phytohormone Production by Trichoderma and Its Impact on Plant Growth

A key mechanism by which *Trichoderma* promotes plant growth is the production of phytohormones that directly influence plant development [11,94]. From an evolutionary perspective, fungi appear to have developed mechanisms to perceive phytohormones as environmental signals. In this context, phytohormones have been reported to induce growth and differentiation processes in fungi themselves [95]. Likewise, the ability to biosynthesize hormones, a trait shared by both pathogenic and symbiotic fungi, has been widely documented [95] and *Trichoderma* is no exception [11]. This capacity to biosynthesize and secrete phytohormones is not limited to host adaptation, representing an important mechanism of chemical communication between *Trichoderma* and its host plant [94,96]. Under this model, fungal phytohormones may act as signals for recruitment and modulation of plant development, promoting root system expansion [97,98]. By promoting plant growth, *Trichoderma* likely increases the availability of plant-derived photoassimilates, which in turn favors fungal colonization and the establishment of a stable niche. This reciprocal exchange reinforces the symbiotic association between both organisms [39] resembling the bidirectional nutrient exchange observed in mycorrhizal symbioses [99].

In particular, *Trichoderma* has been reported to synthesize phytohormones associated with plant development, such as auxins, gibberellins, cytokinins, and abscisic acid, which contribute to plant growth promotion [11,100]. Consistently, genomic analyses have revealed the presence of genes associated with the biosynthesis and signaling of these phytohormones, suggesting that the fungus possesses an intrinsic capacity to both produce and respond to these molecules, thereby contributing to its own physiology and modulating host plant growth [52].

### 4.1. Role of Auxins in the Trichoderma-Plant Interaction

Auxins, particularly indole-3-acetic acid (IAA), are among the best-characterized phytohormones mediating plant-*Trichoderma* interactions. Several *Trichoderma* species have been reported to synthesize IAA through tryptophan-dependent pathways, thereby altering host root development [11,52,100]. However, the production of IAA and other indolic compounds is highly strain-dependent and influenced by culture conditions, potentially contributing to differences in the growth-promoting effects of *Trichoderma* strains [11]. These microbial signals trigger classic auxin-responsive phenotypes in the host, including increased lateral root formation [12,97]. Notably, this phenotype can be induced even in the absence of physical contact through airborne volatile compounds, as demonstrated in *A. thaliana* [12]. Taken together, this evidence indicates that *Trichoderma* acts not only as an exogenous source of auxins but also as a modulator of auxin signaling and root development, remodeling root architecture and enhancing resource acquisition [56,94,97,101].

### 4.2. Ethylene Regulation and Its Role in Root Development

Ethylene (ET) modulation is a key factor in balancing growth and defense during the *Trichoderma*-plant interaction. Another mechanism by which these fungi promote plant growth is through the production of the enzyme ACC deaminase (ACCD), which degrades 1-aminocyclopropane-1-carboxylic acid (ACC), the immediate precursor of ET [102,103]. Silencing the *acdS* gene in *T. asperellum* significantly reduces its ability to promote plant growth, confirming the central role of ACC deaminase in this process [103]. In this context, ACC exuded by roots can be taken up by the fungus in the rhizosphere and metabolized, thereby reducing the availability of this precursor for ET biosynthesis in the plant. Consequently, endogenous ET levels and their inhibitory effects on root growth are reduced, promoting cell elongation and a more efficient root architecture, particularly under stress conditions [102,103]. On the other hand, *T. atroviride* has been reported to biosynthesize ET *in vitro*, and host perception of this fungus-derived hormone involves key components of the ethylene signaling pathway, including Ethylene Insensitive 2 (EIN2) and Constitutive Triple Response 1 (CTR1). The interplay between ethylene and auxin signaling pathways subsequently modulates root system architecture and plant development [104]. Therefore, *Trichoderma*-mediated modulation of ET homeostasis and signaling likely contributes to the regulation of root growth and architecture according to the plant’s physiological status and environmental conditions.

### 4.3. Gibberellins and Cytokinins: Fungal Production and Their Role in Plant Growth

Beyond auxins, *Trichoderma* also produces gibberellins (GAs) and cytokinins (CKs), expanding its hormonal repertoire for plant growth promotion [52,100]. The production of bioactive GAs, particularly GA_1_ and GA_4_, has been reported in several *Trichoderma* species, although their levels vary depending on the strain and growth conditions [52,100]. In wheat under drought stress, high-GA-producing strains such as *T. virens* T49 and *T. harzianum* T115 significantly enhance plant growth, whereas strains producing lower levels of GAs show weaker effects. This variation suggests that GA production contributes to the plant growth-promoting capacity of *Trichoderma* [100]. Gibberellins promote growth by triggering the degradation of DELLA proteins, which repress cell elongation [105]. *Trichoderma* likely enhances this signaling both through direct GA biosynthesis and ACC deaminase activity, which reduces ET levels, a hormone known to stabilize DELLA repressors [102,103,106]. Together, fungal GA production and ET modulation are likely to promote DELLA degradation, ultimately favoring cell elongation and plant development [100,105,106].

Likewise, the production of CKs by *Trichoderma*, mainly as *cis*-zeatin (cZ) and isopentenyladenine (iP) derivatives, has been detected even in axenic cultures, indicating that the fungus possesses an intrinsic CK metabolism. In co-culture with *Arabidopsis*, analyses of several strains showed that *Trichoderma* increases cZ- and iP-type CKs while reducing *trans*-zeatins, thereby modulating the host plant hormonal profile [107]. CK production varies depending on the strain and culture conditions [100]. Moreover, metabolomic analyses revealed the accumulation of CKs in tomato plants treated with *T. koningii* TK7, further supporting the role of this fungus in inducing hormonal changes in the host plant [108].

### 4.4. Plant Hormonal Regulation Mediated by Trichoderma VOCs

In addition to producing phytohormones directly, *Trichoderma* emits VOCs that can induce the biosynthesis and/or accumulation of phytohormones in plants without direct physical contact. For instance, exposure to *Trichoderma* VOCs increases auxin accumulation in roots and promotes lateral root formation [12]. Transcriptomic analyses further support this notion, revealing that *Trichoderma* VOCs reprogram the expression of genes involved in auxin signaling, hormone homeostasis, and root development [81]. Taken together, these findings indicate that *Trichoderma* VOCs act as signaling molecules that regulate hormonal pathways and reshape root architecture. While evidence confirms that *Trichoderma* produces phytohormones and triggers their biosynthesis in plants, the underlying mechanisms remain poorly understood. Integrated functional studies and multi-omics approaches are needed to determine how fungal volatile metabolites induce signaling pathways, reprogram plant hormonal homeostasis, and promote growth.

## 5. Nutrient Acquisition by Trichoderma and Its Impact on Plant Biomass

### 5.1. Phosphate Solubilization and Iron Availability Through Trichoderma Siderophores in Plant Growth

**Figure 2:**
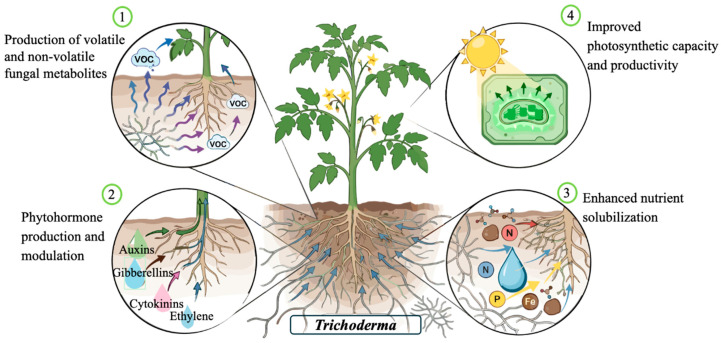
Mechanisms of *Trichoderma*-mediated plant growth and development. (1) Production of volatile and non-volatile fungal metabolites. These compounds mediate plant–fungus communication and modulate plant physiological responses. (2) Phytohormone production and modulation. *Trichoderma* produces and regulates the balance of key hormones, including auxins, gibberellins, cytokinins, and ethylene, thereby influencing plant development and root architecture. (3) Nutrient mobilization and water uptake. *Trichoderma* enhances the availability and acquisition of essential nutrients, including phosphorus, nitrogen, and iron, while improving water uptake. (4) Enhanced photosynthesis and productivity. The interaction with *Trichoderma* enhances plant photosynthetic capacity, resulting in greater biomass accumulation and crop yield. Illustration generated with FigureLabs and subsequently modified and validated by the authors for scientific accuracy.

*Trichoderma* promotes the growth of diverse plant species by increasing nutrient bioavailability and uptake (Figure 2) [109,110]. This effect is associated with changes in rhizosphere pH resulting from acidification induced by plasma membrane H^+^-ATPase activity and the production of organic acids by the fungus, mechanisms that favor nutrient solubilization and plant uptake [90].

The phosphate-solubilizing capacity of *Trichoderma* varies among species. For example, *T. koningiopsis* solubilizes calcium phosphates through rhizosphere acidification mediated by the production of organic acids, mainly citric and oxalic acids [109]. In contrast, *T. harzianum* T-22 employs complementary mechanisms, such as cation chelation and redox activity, allowing it to mobilize insoluble phosphorus even in neutral or alkaline soils, where acidification is less efficient [90]. Recently, the strain *T. lixii* ORT2 was reported to solubilize insoluble phosphates, including tricalcium phosphate and zinc and iron phosphates, through the coordinated expression of alkaline phosphatase and siderophores. This improves phosphorus availability for chickpea (*Cicer arietinum*), stimulating plant growth and reducing the dependence on chemical fertilizers [111].

Several *Trichoderma* species secrete siderophores that chelate and mobilize iron in the rhizosphere, making it accessible for plant uptake (Figure 2). For example, Kabir & Bennetzen [112] found that *T. harzianum* releases siderophores that bind Fe (III). This complex enhances iron absorption in sorghum roots via specific transporters such as SbYS1, thereby significantly improving iron bioavailability under high-pH conditions [112]. Similarly, the T6 strain of *T. asperellum* produces elevated levels of siderophores and organic acids; these metabolites not only mobilize iron but also facilitate the reduction of Fe^3+^ to Fe^2+^ via Fe^3+^-chelate reductase enzymes, promoting nutrient assimilation and growth in cucumber [113]. Likewise, the XZ11-1 strain of *T. virens* produces siderophores that increase iron availability and suppress the growth of *F. oxysporum* by competing for this micronutrient through chelation, thereby promoting growth in banana plants [114].

In addition to their role in nutrient mobilization, siderophores produced by *Trichoderma* have been associated with hormonal regulation in plants. Zhao et al. [115] found that siderophores produced by *T. asperellum* Q1 contribute to iron mobilization and also correlate with increased auxin levels in *Arabidopsis*, even in the absence of the fungus, thereby promoting root development and nutrient uptake [115]. Similarly, Eslahi et al. [116] observed that recombinant strains of *T. harzianum* carrying a modified *chit42* gene (fused to a chitin-binding domain), which exhibit higher siderophore production, also show increased auxin secretion, which is associated with improved root colonization and enhanced growth in bean plants [116].

Overall, *Trichoderma* enhances the bioavailability of phosphorus and iron through mechanisms such as medium acidification, phosphatase-mediated hydrolysis, and siderophore-mediated chelation, thereby promoting plant growth and reducing dependence on chemical fertilizers.

### 5.2. Influence of Trichoderma on Nitrogen Assimilation and Regulation of Plant Transporters

*Trichoderma* colonizes the rhizosphere and promotes root development, thereby enhancing nitrogen uptake and assimilation from nitrate (NO_3_^−^) and ammonium (NH_4_^+^) (Figure 2). This effect is associated with the activation of key enzymes involved in nitrogen metabolism, such as nitrate reductase, and the induction of nitrate transporters in roots, which improve nitrogen use efficiency (NUE) and plant growth even under low nitrogen availability [117,118]. Singh et al. [117] demonstrated that *T. asperellum* T42 enhances NUE in tobacco by activating nitrate reductase activity and nitric oxide (NO) production, thereby promoting the development of lateral roots and root hairs. In addition, this strain induces the expression of the nitrate transporters *NRT2.1* and *NRT2.2*, resulting in greater nitrate uptake [117]. Likewise, in pea (*Pisum sativum* L.), T42 improved NUE and root growth through NO production, induction of nitrate transporters, and repression of the ammonium transporter *AMT1.1*, thus promoting preferential nitrate uptake [119].

Overall, *Trichoderma* enhances nitrogen acquisition and assimilation by modulating enzymatic activities, nitrate transporters, and NO signaling, thereby improving NUE and promoting plant growth [118].

### 5.3. Trichoderma-Induced Chlorophyll and Sugar Accumulation

*Trichoderma* can improve plant photosynthetic performance by increasing chlorophyll content, enhancing sugar accumulation, and promoting biomass production (Figure 2) [120,121]. Root colonization by endophytic *Trichoderma* strains increases photosynthetic pigment levels and activates antioxidant defenses, reducing ROS accumulation and protecting the photosynthetic machinery under stress conditions. As a result, CO_2_ assimilation is improved, leading to greater plant growth and productivity [53].

Exposure of *A. thaliana* to VOCs emitted by *T. atroviride*, *T. virens*, and *T. asperellum* increased chlorophyll content and biomass, demonstrating that these compounds improve photosynthetic efficiency and promote plant growth. In contrast, VOCs produced by *T. reesei*, a predominantly saprophytic species, did not modify these parameters compared to the control [11], suggesting that plant-associated *Trichoderma* species produce specific VOCs involved in plant biostimulation. Likewise, Kabir et al. [122] found that inoculation of soybean plants with *T. harzianum* T22 under iron deficiency increased chlorophyll content and photosynthetic efficiency. This effect was associated with enhanced iron mobilization and uptake, which are essential for chlorophyll synthesis and photosystem function, as well as with the activation of antioxidant mechanisms that protect the photosynthetic apparatus from oxidative stress under micronutrient-limiting conditions [122].

In addition, Liu et al. [123] reported that inoculation with *T. viride* kf57 increased chlorophyll content, antioxidant enzyme activities (peroxidase and superoxide dismutase), nitrate reductase activity, and soluble sugar levels (sucrose and reducing sugars) in melon grown under monoculture soil conditions [123]. These physiological changes were correlated with improved carbon metabolism and higher crop yield.

Overall, *Trichoderma* boosts plant photosynthetic performance by mobilizing iron, activating antioxidant defenses, and producing bioactive metabolites. These mechanisms also improve phosphorus and nitrogen uptake, increasing biomass and productivity, while enhanced CO_2_ assimilation may potentially contribute to climate change mitigation.

## 6. Agricultural Applications and Future Perspectives of Trichoderma

*Trichoderma* species are among the most widely studied and utilized beneficial fungi in agriculture due to their ability to promote plant growth, control phytopathogens, and mitigate biotic and abiotic stresses [53]. However, challenges remain in maximizing their effectiveness under field conditions, including adaptation to diverse edaphoclimatic environments, compatibility with agrochemicals, and the development of microbial consortia with other beneficial microorganisms [124,125].

### 6.1. Formulations and Application Technologies

In agriculture, *Trichoderma* strains are used as bioinoculants via seed treatment, soil application, and foliar sprays to control aerial pathogens [125,126]. Their effectiveness depends on their ability to establish in the rhizosphere and maintain a stable interaction with the host plant. The success of these bioinputs is closely linked to formulation, as it determines spore viability, soil persistence, and root colonization capacity [125,127]. Commercial production of *Trichoderma*-based bioproducts primarily relies on conidia due to their ease of mass production, although some formulations also incorporate chlamydospores, which exhibit greater tolerance to environmental stress and a longer shelf life [91]. To preserve fungal propagule viability and improve bioproduct stability, solid carriers or liquid matrices are used in combination with technologies such as freeze-drying, spray-drying, and microencapsulation, which protect inoculants against adverse conditions [127,128,129]. Mineral supports can further enhance these strategies by providing physical protection and promoting microbial establishment after soil application [127]. These approaches enable the development of stable products such as wettable powders, granules, and liquid suspensions that can be applied to seeds, soil, or irrigation systems, thereby expanding the agronomic versatility of *Trichoderma* [126].

*Trichoderma* can reshape the rhizosphere microbiome by modulating microbial community assembly and niche availability, thereby enhancing root colonization and plant performance [130]. Native microorganisms may compete for nutrients and colonization sites, limiting *Trichoderma* establishment [7]. However, synergistic interactions with PGPR, such as *Bacillus* and *Pseudomonas* spp., can facilitate fungal colonization while enhancing plant growth and tolerance to abiotic stress [7,131]. Moreover, several *Trichoderma*–bacteria microbial consortia have been developed to improve bioinoculant efficacy [7,132]. These consortia enhance nutrient availability, phytohormone production, and plant defense activation, making them a promising strategy for improving crop performance under agricultural conditions [7,133].

### 6.2. Challenges in the Production and Formulation of Trichoderma in Agriculture

Despite its broad agronomic potential, the commercial implementation of *Trichoderma* faces challenges related to production scalability, formulation stability, regulatory frameworks, and field performance, as laboratory results are not always reproduced under field conditions due to strain specificity, crop genotype, soil properties, and environmental factors [124,126,134]. Therefore, large-scale production requires optimization of fermentation processes to ensure high levels of viable spores, as their viability and performance can be affected by temperature, desiccation, UV radiation, and formulation type [135]. Although technologies such as encapsulation, carrier optimization, and drying have improved product stability, many commercial bioinputs still exhibit limited shelf life and reduced viability under field conditions, affecting colonization, metabolite production, and agronomic performance [127,136]. In this context, encapsulation in nanocellulose-based matrices has emerged as a promising strategy to extend shelf life and protect *Trichoderma* from heat stress, UV radiation, and fungicide exposure [137]. Furthermore, compatibility with agrochemicals remains a significant limitation, as certain fungicides and fertilizers can adversely affect the survival and efficacy of *Trichoderma* under field conditions [124]. Accordingly, the development of formulations compatible with conventional agricultural practices, together with delivery, protection, and controlled-release systems, represents a key strategy to improve the stability, efficacy, and adoption of *Trichoderma*-based bioinputs in sustainable agriculture.

### 6.3. Future Perspectives of Trichoderma-Based Bioinputs

Molecular biology studies, particularly those based on omics approaches, are enabling a more precise understanding of *Trichoderma*-plant interactions [138]. This knowledge facilitates the selection and development of strains with greater specificity and agronomic efficiency. Furthermore, genomic and metabolomic studies are accelerating the identification of bioactive metabolites in *Trichoderma*, expanding their potential for biocontrol and the development of sustainable agricultural bioinputs [139,140]. In addition, integrating *Trichoderma*-based bioinputs into precision agriculture systems could optimize their application based on crop and environmental conditions, thereby improving agronomic performance [124]. Likewise, the development of microbial consortia integrating *Trichoderma* with other compatible microorganisms represents a promising strategy to improve phytopathogen control, promote plant growth, and increase crop productivity [141]. Future studies should integrate precision agriculture tools with information on soil properties, climatic conditions, and crop genotypes to select strains and design microbial consortia adapted to specific agroecosystems.

## 7. Conclusions

*Trichoderma* promotes plant growth through diverse mechanisms, including metabolite production, hormonal modulation, enhanced nutrient acquisition and bioavailability, and increased photosynthetic capacity. Collectively, these mechanisms contribute to plant growth, productivity, and tolerance to biotic and abiotic stress, highlighting the multifaceted role of *Trichoderma* as a plant growth-promoting microorganism and modulator of plant stress responses. Currently, *Trichoderma* is a key component in the development of agricultural bioinputs; however, challenges remain regarding formulation stability, efficacy, consistency under field and greenhouse conditions, and the scalability of production and application. Furthermore, an important aspect is the marked functional diversity among *Trichoderma* species and strains, since their beneficial traits, including plant growth promotion and biological control, largely depend on the strain genotype, the host plant, and the environmental conditions. Addressing these limitations requires a deeper understanding of strain–plant–environment interactions, as well as the molecular mechanisms underlying the specific functional traits of individual isolates. In addition, technologies that improve microbial establishment and persistence in agricultural systems must be developed.

Leveraging next-generation biotechnology, including omics-based strain selection, advanced formulation technologies, microbial consortia, and precision agriculture tools, will be essential to unlock the full potential of *Trichoderma*. Together, these advances will enhance the reliability and efficacy of *Trichoderma*-based bioinputs, supporting the transition toward more sustainable, resilient, and productive agricultural systems.

## Data Availability

All data supporting the findings of this review are available within the references cited in this manuscript.
